# Molecular evolution of dihydrouridine synthases

**DOI:** 10.1186/1471-2105-13-153

**Published:** 2012-06-28

**Authors:** Joanna M Kasprzak, Anna Czerwoniec, Janusz M Bujnicki

**Affiliations:** 2 Institute of Molecular Biology and Biotechnology, Adam Mickiewicz University, Umultowska 89, PL-61-614, Poznan, Poland; 1 Laboratory of Bioinformatics and Protein Engineering, International Institute of Molecular and Cell Biology, Trojdena 4, PL-02-109, Warsaw, Poland

**Keywords:** Dihydrouridine synthases, Protein structure prediction, Fold recognition, Remote homology, RNA modification, Molecular evolution, Enzymes acting on RNA

## Abstract

**Background:**

Dihydrouridine (D) is a modified base found in conserved positions in the D-loop of tRNA in Bacteria, Eukaryota, and some Archaea. Despite the abundant occurrence of D, little is known about its biochemical roles in mediating tRNA function. It is assumed that D may destabilize the structure of tRNA and thus enhance its conformational flexibility. D is generated post-transcriptionally by the reduction of the 5,6-double bond of a uridine residue in RNA transcripts. The reaction is carried out by dihydrouridine synthases (DUS). DUS constitute a conserved family of enzymes encoded by the orthologous gene family COG0042. In protein sequence databases, members of COG0042 are typically annotated as “predicted TIM-barrel enzymes, possibly dehydrogenases, nifR3 family”.

**Results:**

To elucidate sequence-structure-function relationships in the DUS family, a comprehensive bioinformatic analysis was carried out. We performed extensive database searches to identify all members of the currently known DUS family, followed by clustering analysis to subdivide it into subfamilies of closely related sequences. We analyzed phylogenetic distributions of all members of the DUS family and inferred the evolutionary tree, which suggested a scenario for the evolutionary origin of dihydrouridine-forming enzymes. For a human representative of the DUS family, the hDus2 protein suggested as a potential drug target in cancer, we generated a homology model. While this article was under review, a crystal structure of a DUS representative has been published, giving us an opportunity to validate the model.

**Conclusions:**

We compared sequences and phylogenetic distributions of all members of the DUS family and inferred the phylogenetic tree, which provides a framework to study the functional differences among these proteins and suggests a scenario for the evolutionary origin of dihydrouridine formation. Our evolutionary and structural classification of the DUS family provides a background to study functional differences among these proteins that will guide experimental analyses.

## Background

**Figure 1:**
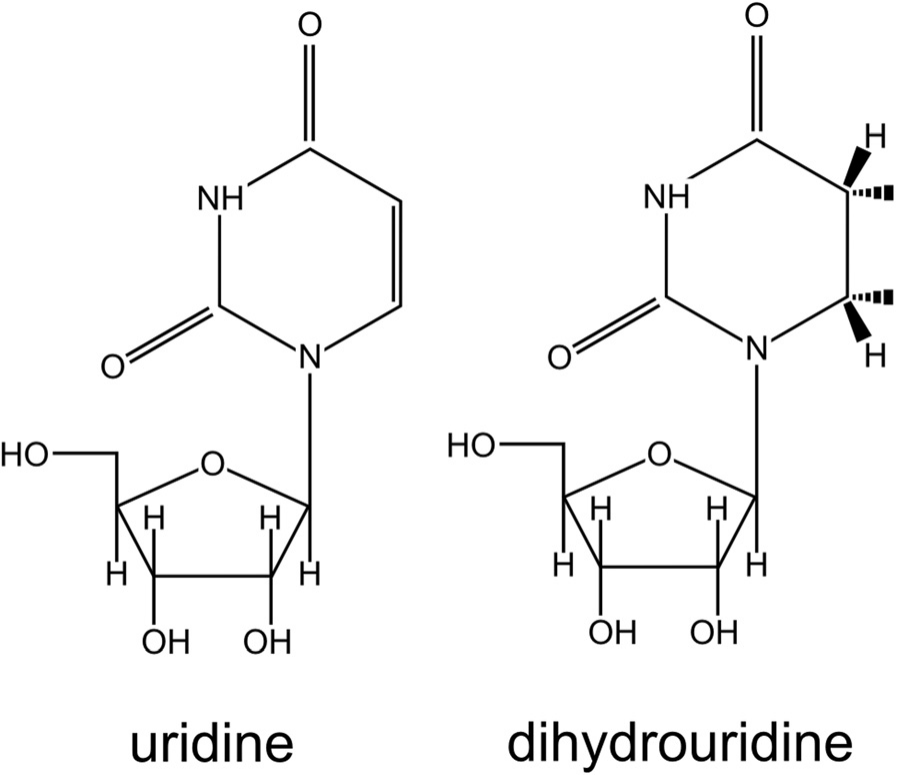
Chemical structures of uridine (U) and dihydrouridine (D).

Dihydrouridine (D; 5,6-dihydro-uridine) is one of the posttranscriptionally modified nucleosides. It is a product of the reduction of uridine (U), and can be further modified to 5-methyldihydrouridine (m5D). D is commonly present in the tRNA from Bacteria, Eukaryota, and some Archaea [1]. It was identified in six positions in the “D-loop” of the tRNA (16, 17, 20a, 20b) and in position 47 in the variable loop. A single D is present in the central loop of domain V in *Escherichia coli* 23 S ribosomal RNA [2]. D is unique among modified nucleosides in possessing a C5-C6 single bond rather than the usual C5-C6 double bond (Figure 1). Compared with U, D is not planar and its ring is not aromatic, which hampers the ability to form stacking interactions with other nucleosides. Interactions and loop formation must be simultaneously accommodated. NMR analyses showed that D may destabilize the structure of tRNA by promoting the C2′-endo conformation of the sugar moiety instead of C3′-endo, which is thermodynamically more preferred [3]. A conformational change caused by this modification probably increases flexibility and dynamics of RNA regions that participate in 3D interactions. Consequently, D occurs mainly in single stranded loops and in regions of RNA with high tension in the nucleotide chain [4]. Recent studies have shown that the level of D is elevated in tumor cells [5]. D is also common in psychrophilic bacteria under conditions where thermal motion, enzymatic reaction rates and interactions between biomolecules are adjusted to low temperatures [3]. However, the knowledge of the exact role of D in mediating RNA function is still limited.

D is introduced in the RNA by dihydrouridine synthases (DUS). They constitute a conserved family of enzymes encoded by the orthologous gene family COG0042. In protein sequence databases, members of COG0042 are typically annotated as “predicted TIM-barrel enzymes, possibly dehydrogenases, nifR3 family”. They are found in all free-living organisms whose genomes have been sequenced. For instance, in *E. coli* three DUS have been identified (DusA, DusB, DusC), while *Saccharomyces cerevisiae* has four members of this family (Dus1p, Dus2p, Dus3p, Dus4p) [6] (see the most up to date list of enzymes in the MODOMICS database [7]). All yeast DUS are substrate specific and can modify only one or two positions in a tRNA molecule. It was shown [8] that Dus1p and Dus4p are able to modify two different positions in tRNA, U16/U17 and U20a/U20b_,_ respectively. The other two yeast DUS, Dus2p and Dus3p, catalyze modification of unique positions in tRNA: 20 and 47, respectively. The targets of Dus1p, Dus2p, and Dus4p are within the D-loop of tRNA, while Dus3p acts on residues within a variable loop [6,9]. Among the three DUS identified in *E. coli*, it has been reported that DusA (formerly YjbN) acts on position 21 of tRNA [6]. It has been noticed that while the *Mycoplasma mycoides* genome encodes only a single DUS, its tRNAs contain D at many different positions [10].

Dihydropirimidine dehydrogenase (DHPDH) [11] and dihydroorotate dehydrogenase (DHODH) [12] are enzymes with activities similar to DUS. DHPDH catalyzes NADPH-dependent reduction of uracil to 5,6-dihydrouracil, while DHODH catalyzes oxidation of dihydroorotate to orotate in the pirimidine biosynthesis pathway. Both enzymes are homologous to DUS, their crystallographic structures have been determined and the mechanism of action is known. The availability of these structures prompted us to use comparative modeling to elucidate the structure of the DUS active site, its interactions with the substrate, and the mechanism of action.

Studies on DUS enzymes may have an applied edge, as it has been found that a human member of the DUS family, hDus2, has been implicated in cancer. In particular, the Nakamura group discovered that silencing the *HDUS2* gene decreases the abundance of D residues in tRNA molecules, reduces the effectiveness of translation, and in consequence blocks the growth of cancer cells [5]. This finding suggests that hDus2 may be a target for anticancer therapy. In particular, selective inhibitors able to block the DUS activity of hDus2 by interactions with its active site or tRNA binding site may be interesting leads for new anti-cancer drugs.

At the time of the original submission of this article (21^st^ June 2011) the structure of DUS enzymes was unknown. To elucidate sequence-structure-function relationships in the DUS family we carried out a comprehensive bioinformatic analysis, including sequence searches, clustering and phylogenetic analyses, and protein structure prediction. We have also built a homology model of hDus2 and predicted enzyme-substrate interactions. Based on these analyses, we identified a putative active site and substrate-binding residues and proposed the mechanisms of DUS activity and its potential inhibition. While the manuscript was under review, a crystal structure of another representative of the DUS family has been determined in complex with tRNA [13], giving us an opportunity to validate theoretical predictions.

## Results and discussion

### Sequence database searches and retrieval of members of the DUS family

To identify a complete set of DUS sequences, we used full-length sequences of experimentally characterized DUS from *E. coli* and *S. cerevisiae* to carry out exhaustive PSI-BLAST [14] searches of the (nr) database (until reaching convergence) and retrieved all sequences reported with e-value better than 1e-25. We removed identical proteins retrieved in different searches and, as a result, we obtained a set of about 11000 sequences.

### Subdivision of DUS sequences into closely related groups

Clustering of all full-length DUS sequences was performed based on their pair-wise BLAST similarity scores, using CLANS [15]. We had experimentally found that for this particular dataset the P-value threshold of 1e-7 produced qualitatively best results (more stringent values caused disconnection of the most diverged families, while more permissive values caused over-compaction of the whole dataset into a single cluster with only a few outliers). This clustering revealed that from all retrieved sequences only members of COG0042 and KOG2333, KOG2334 and KOG2335 grouped together with the genuine DUS proteins. All other gathered sequences showed only slight similarity to DUS and while they should be considered homologous, they are likely to exhibit different enzymatic activities, possibly based on a generally similar mechanism. Importantly, the clustering confirmed sequence similarity of KOG1799 (dihydropirimidine dehydrogenase) and KOG1436 (dihydroorotate dehydrogenase) to DUS (data not shown). This analysis has also confirmed that the TM0096 protein from *Thermatoga maritima*, annotated as “a putative flavin oxidoreductase”, whose crystal structure has been solved in complex with a flavin mononucleotide by the Structural GenomiX consortium (Protein Data Bank ID - 1vhn) [16], is indeed a member of the DUS family.

**Figure 2:**
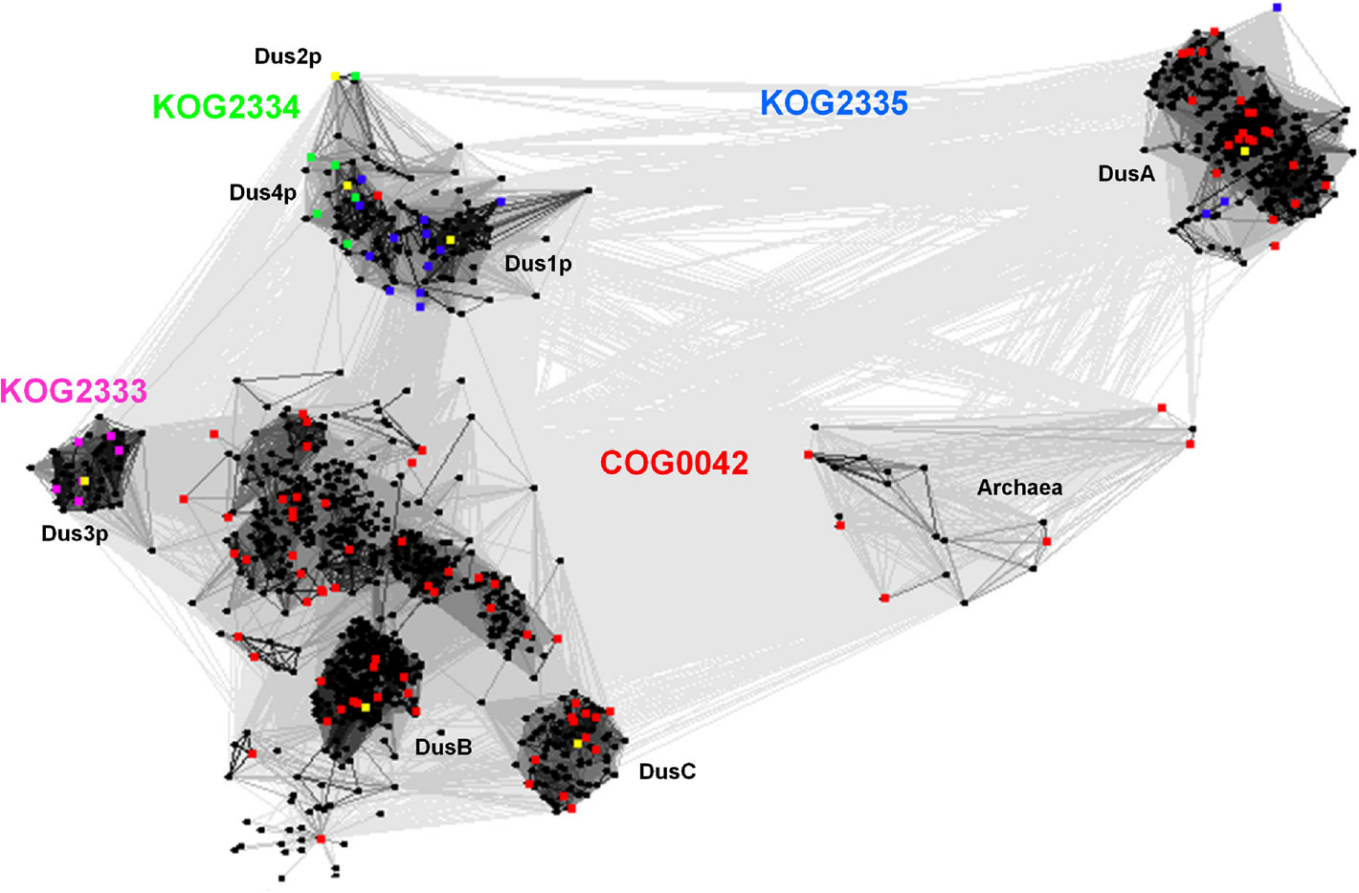
**Two-dimensional projection of the CLANS clustering results obtained for full-length sequences of all DUS family members.** Proteins are indicated by dots, members of COGs and KOGs are colored (COG0042 members are shown in red, KOG2333 in magenta, KOG2334 in light green and KOG2335 in blue). Representative members of each subfamily (Dus1p, Dus2p, Dus3p, Dus4p, DusA, DusB and DusC) are indicated as yellow dots. Lines indicating sequence similarity detectable with BLAST are colored by a different shades of grey according to the BLAST P-value (black: P-value < 10^-200^, light grey: P-value < 10^-5^).

To identify relationships between sequences within the DUS family, we took only true DUS members including COG0042, KOG2333, KOG2334 and KOG2335 sequences and reclustered them (Figure 2). We have experimentally found that for this particular dataset the P-value threshold of 1e-4 produced qualitatively best results. This simple clustering produced a clear-cut separation of most original COGs and their close homologs found by PSI-BLAST into 8 distinct clusters: archaeal Dus, DusA, DusB, DusC, Dus1, Dus2, Dus3 and Dus4. Archaeal Dus is a small group of proteins, relatively diverged from each other, and relatively remotely related to all other groups. DusA is a group of mainly plant and bacterial enzymes. DusA members show high sequence similarity to each other, but are very different from all the other groups, which suggests that they had evolved rapidly. Three other clusters: Dus1, Dus2, and Dus4 are relatively closely related to each other; they group together fungal, animal and plant proteins. Finally, groups Dus3, DusB, and DusC are closely related to each other. Dus3 contains eukaryotic sequences, whereas DusC has only bacterial members.

Based on the results of preliminary clustering, we extracted members of individual subfamilies, calculated family-specific multiple sequence alignments using ClustalX [17] and adjusted them manually (as described in Methods) to remove truncated sequences and redundant, nearly identical versions of the same protein, and to improve the placement of insertions and deletions. We have submitted these alignments, as well as individual sequences of representatives from *E. coli* and *S. cerevisiae* to the GeneSilico metaserver [18], to predict a number of structural and functional features, such as: secondary structure, intrinsic disorder, RNA-binding residues, and to identify matches to related proteins with experimentally determined structures, in particular to TM0096.

### A multiple sequence alignment of representative DUS sequences

**Figure 3:**
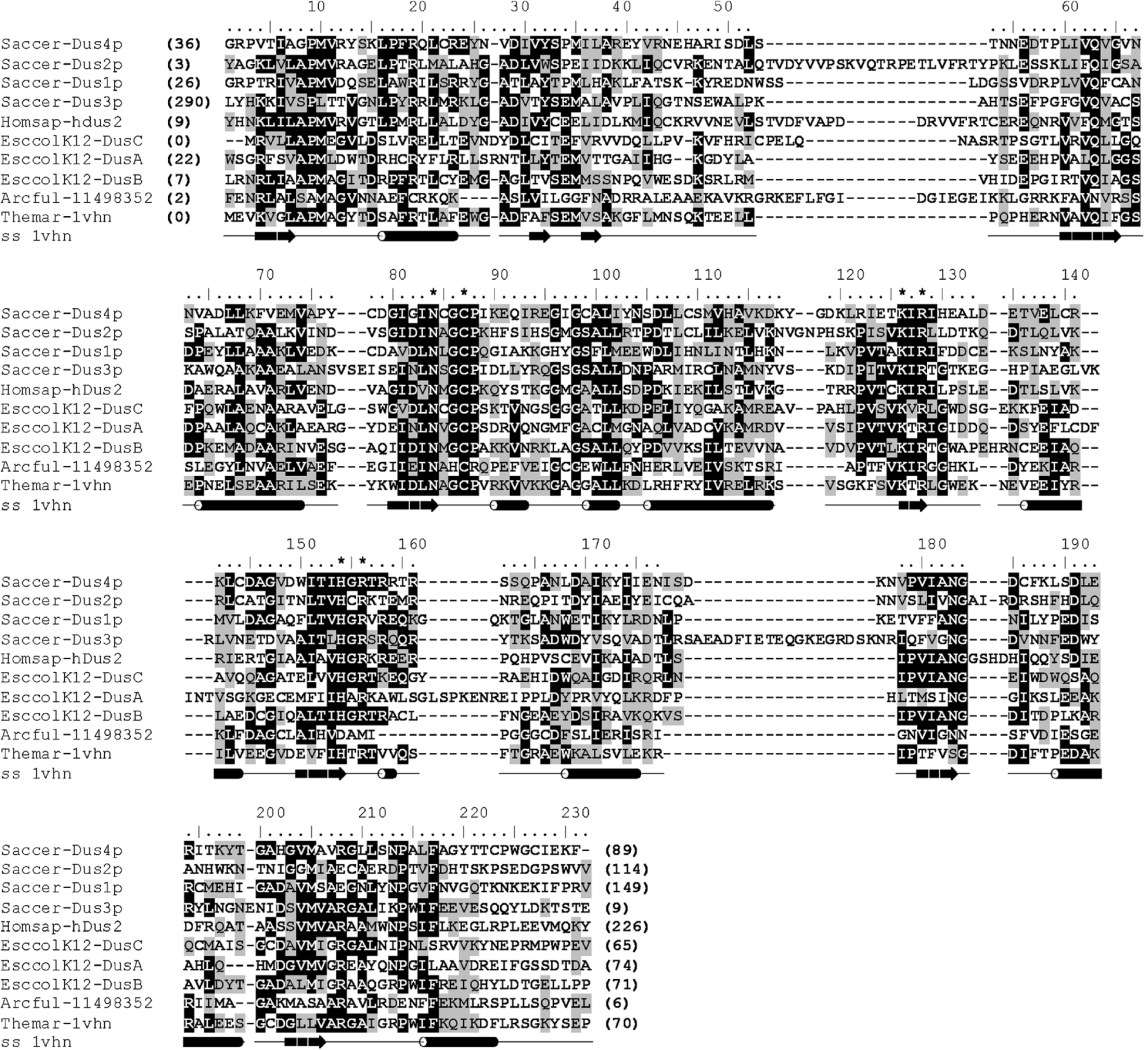
**Multiple sequence alignment of selected representatives of DUS subfamilies.** Sequences are denoted by species’ name (six-letter abbreviation for genus or species e.g. Bacsub for *B. subtilis*), followed by protein name (if assigned) or the PDB code or NCBI Gene Identification (GI) number. The variable termini and non-conserved insertions are not shown; the number of omitted residues is indicated in parentheses. Secondary structure elements observed in the crystal structure of TM0096 (PDB code 1vhn) are shown under the group alignment as tubes (helices) and arrows (strands). Invariant and conserved residues are highlighted in black and grey, respectively. The potential catalytic residues are indicated by an asterisk (“*”).

A multiple sequence alignment of DUS sequences (full alignment available as Additional file 1, representatives shown in Figure 3) was generated based on alignments of individual subfamilies to the TM0096 structure, produced by protein fold-recognition methods (for more detailed information see Methods). There are three motifs conserved to some extent between all DUS subfamilies that include residues found to be important for the catalysis in *E. coli* DusA [10]. Motif NXGCP (positions 83–88 in the alignment; X indicates any residue) is highly conserved within all subfamilies and contains the Cys residue which is absolutely indispensable for activity in *E. coli* DusA (C114). In archaeal DUS, G is substituted by H, and P by K or R. The second conserved motif KXR (positions 126–128 in the alignment) contains the Lys residue, which is indispensable for catalysis in *E. coli* DusA (K153). The third conserved motif HXR (positions 154–156 in the alignment) contains further charged residues likely to be involved in catalysis. In archaeal DUS, the R residue in this motif is replaced by D. The conserved residues of these motifs are located in the C-termini of β-strands located in the core of the experimentally determined structure of the TM0096 protein (and predicted to be conserved in all members of the DUS family).

The multiple sequence alignment also reveals residues that are specific to individual subfamilies, and hence may contribute to differences in substrate specificity between subfamilies. For example the eukaryotic Dus1 and Dus4 subfamilies possess common conserved residues (T/S)PM (see positions 50–52 in Additional file 1), which correspond to a very similar motif (T/S)EM present in all bacterial DUS. This region is not conserved in archaeal Dus, Dus2 and Dus3 subfamilies. DusA members possess a subfamily-specific conserved insertion GLSPKENREIPPLD (positions 292 to 336 in Additional file 1). Sequences of Dus3 members are distinguished by a highly conserved motif LTTVGNPPFRR (positions 22–32 in Additional file 1). Further, a common feature of Dus4, DusA, DusC and archaeal Dus is the lack of a conserved GA motif (positions 37–38 in Additional file 1). There are also several regions in the alignment corresponding to fragments with high sequence variability that are enriched in predicted intrinsic disorder and in predicted RNA-binding residues (e.g. positions 55–115, 260–270 in Additional file 1). While the presence or absence or length of these regions may be characteristic for individual subfamilies, their sequences are not highly conserved, which suggests that they may be responsible for sequence-nonspecific interactions with the RNA (e.g. via the backbone). Such regions may for instance influence the subfamily-specific recognition of entire regions in the RNA substrates and restrict the action of enzymes to particular loops in the RNA. Such regions are however typical for eukaryotic sequences, and are relatively scarce in bacterial subfamilies. Nonetheless, we predict that it is not the variability of residues in conserved motifs, but rather the length and sequence composition of loops immediately following the conserved motifs (and shaping the surface around the active site) that may contribute most to the substrate specificity of different DUS members. This prediction remains to be tested experimentally.

### Domain architecture of DUS members

DusB and DusC consist only of the catalytic DUS domain. However, many members of the DUS family possess additional domains or extensions fused to N- or C-termini of the catalytic domain. Dus1p, Dus2p, Dus4p and DusA have additional disordered regions in at least one of the termini. Dus3p contains two long disordered fragments and additionally one zinc finger domain in the N-terminus. Human Dus2 (hDus2) contains a dsRBD (double stranded RNA-binding motif domain) in the C-terminus. The dsRBD domain is also present in Dus2 from other animals, including mammals (e.g. cow, mouse, rat), amphibia (e.g. western clawed frog), flatworms (e.g. blood fluke), nematodes (e.g. filarial nematode worm), and insects (e.g. african malaria mosquito, fruit fly). On the other hand it is absent in Dus2 from fungi and plants. It will be interesting to determine whether the presence of the dsRBD domain may influences the range of substrates modified by enzymes in the Dus2 family (e.g. in the human vs the yeast enzyme).

Eukaryotic proteins often differ from their bacterial counterparts by possessing additional regions that exhibit intrinsic structural disorder, and according to our predictions, DUS proteins are no exception to this rule. Among DUS from *E. coli*, only DusA contains a short N-terminal extension that is predicted to be disordered. Dus1p from *S. cerevisiae* has two regions predicted to be disordered, spanning residues 1–15 and 324–423. Dus2p possesses a C-terminal disordered region spanning residues 330–382. Dus3p has very long disordered fragments in both termini, spanning residues 1–280 and 601–668. Both termini of Dus4p are predicted to be disordered, spanning residues 1–36 and 300–367. Disordered regions are commonly involved in interactions with other molecules, and in DUS they may play a role in tRNA binding and substrate specificity. Computational prediction of RNA binding sites for all DUS representatives from *S. cerevisiae* and *E. coli* (See Methods for details) indicate that disordered regions in Dus1p (residues 356–423), Dus2p (345–382), Dus3p (1–30, 65–90, 145–165, and 655–668), Dus4p (1–36, 335–367), and DusA (1–28) are rich in RNA binding residues.

### Phylogenetic distribution of members of the DUS family

**Figure 4:**
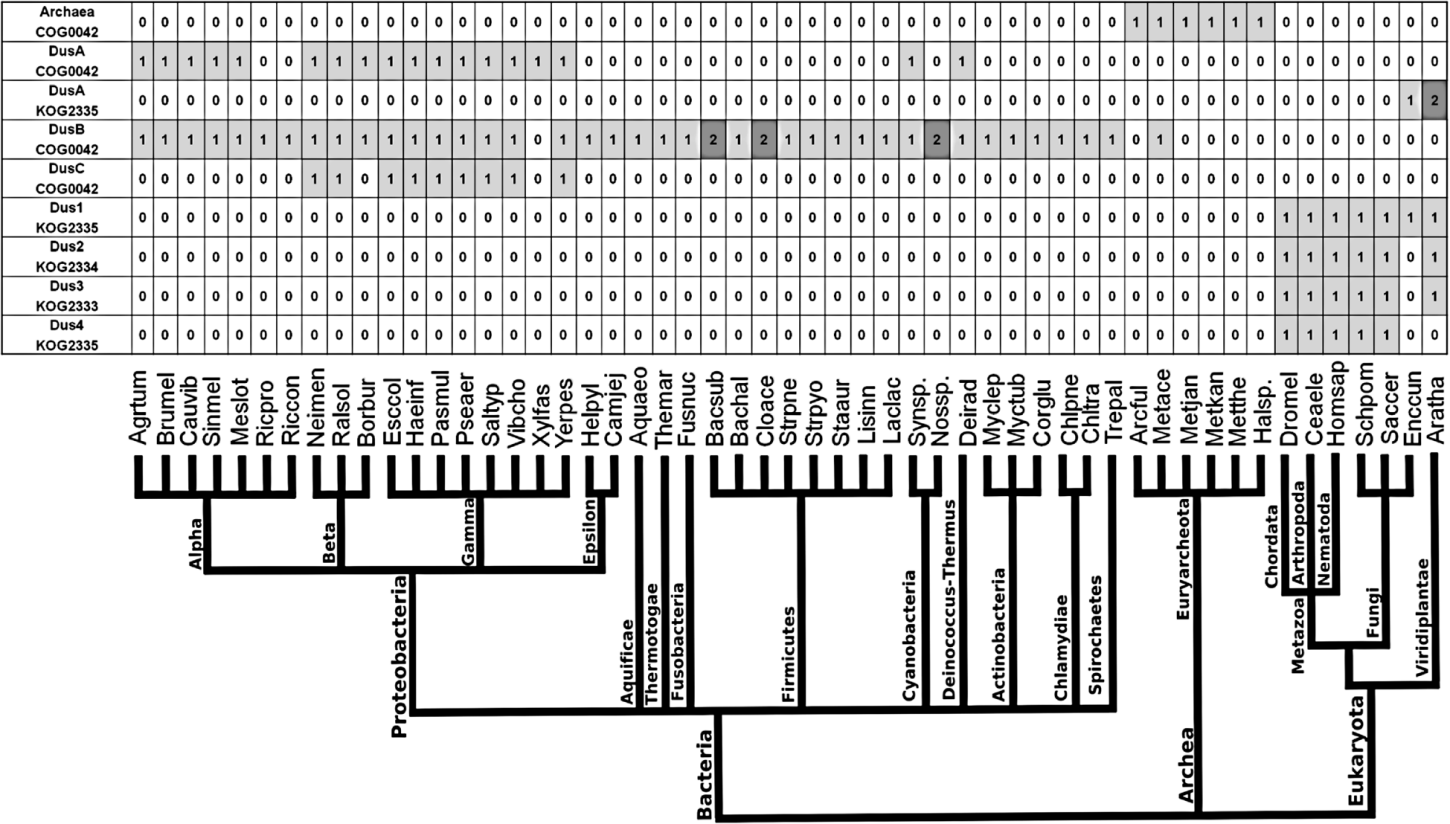
**Phylogenetic distribution of DUS.** DUS sequences have been divided into subfamilies (shown in rows) based on the clustering analysis reported in this article. Columns indicate archaeal, bacterial, and eukaryotic species as given by the COG/KOG database labeled by the abbreviation genus and species name (e.g. Bacsub for *B. subtilis*). Main taxons have been indicated below the plot in the form of a simplified phylogenetic tree. Numbers and shades of grey indicate the number of members in a given genome.

Figure 4 shows a phylogenetic distribution of members of the DUS family with respect to a selected set of completely sequenced genomes in the COG/KOG database (edition 2010) [19]. No subfamily contains members from all major taxa in all three Domains of Life. Archaeal members (all from COG0042) are found only in Euryarcheota. The DusA subfamily includes members of both COG0042 and KOG2335 that are found in nearly all Proteobacteria and Viridiplantae. On the other hand, the only fungal member is from *Encephalitozoon cuniculi*. The DusB subfamily is the most diverse and consists only of members of COG0042. These proteins are found in most Bacteria and among Archaea they are represented by *Methaosarcina acetivorans str. C2A*. DusC is a small subfamily with members of COG0042 from beta- and gamma- Proteobacteria*.* Subfamilies Dus1, Dus2, Dus3 comprise eukaryotic proteins typically found in Metazoa, Fungi and Viridiplantae whereas Dus4 occurs in Metazoa and Fungi but not in Viridiplantae.

### Phylogenetic tree of DUS family

Based on the sequence alignment of the structurally conserved (and therefore reliably alignable) region of DUS sequences (Additional file 1), we attempted to infer the phylogenetic tree of the family. Traditional methods for phylogenetic reconstruction based on sequence data, including neighbor-joining (NJ), minimum evolution (ME), and maximum parsimony (MP) failed to produce a confident tree with well-resolved branches (data not shown). This failure was most likely caused by high sequence divergence and uneven rates of evolution between and within different DUS subfamilies. On the other hand, the number of sequences in the alignment is relatively large for the computationally demanding maximum likelihood (ML) method. Therefore, we decided to use the Bayesian approach, which combines relative reliability of ML with fast scanning of the parameter landscape by the Markov chain Monte Carlo (MCMC) approach implemented in MrBayes3 program [20]. The family trees were calculated for an alignment comprising only 107 representatives selected from gathered COG and KOG sequences. Only well aligned regions were used for the calculations.

**Figure 5:**
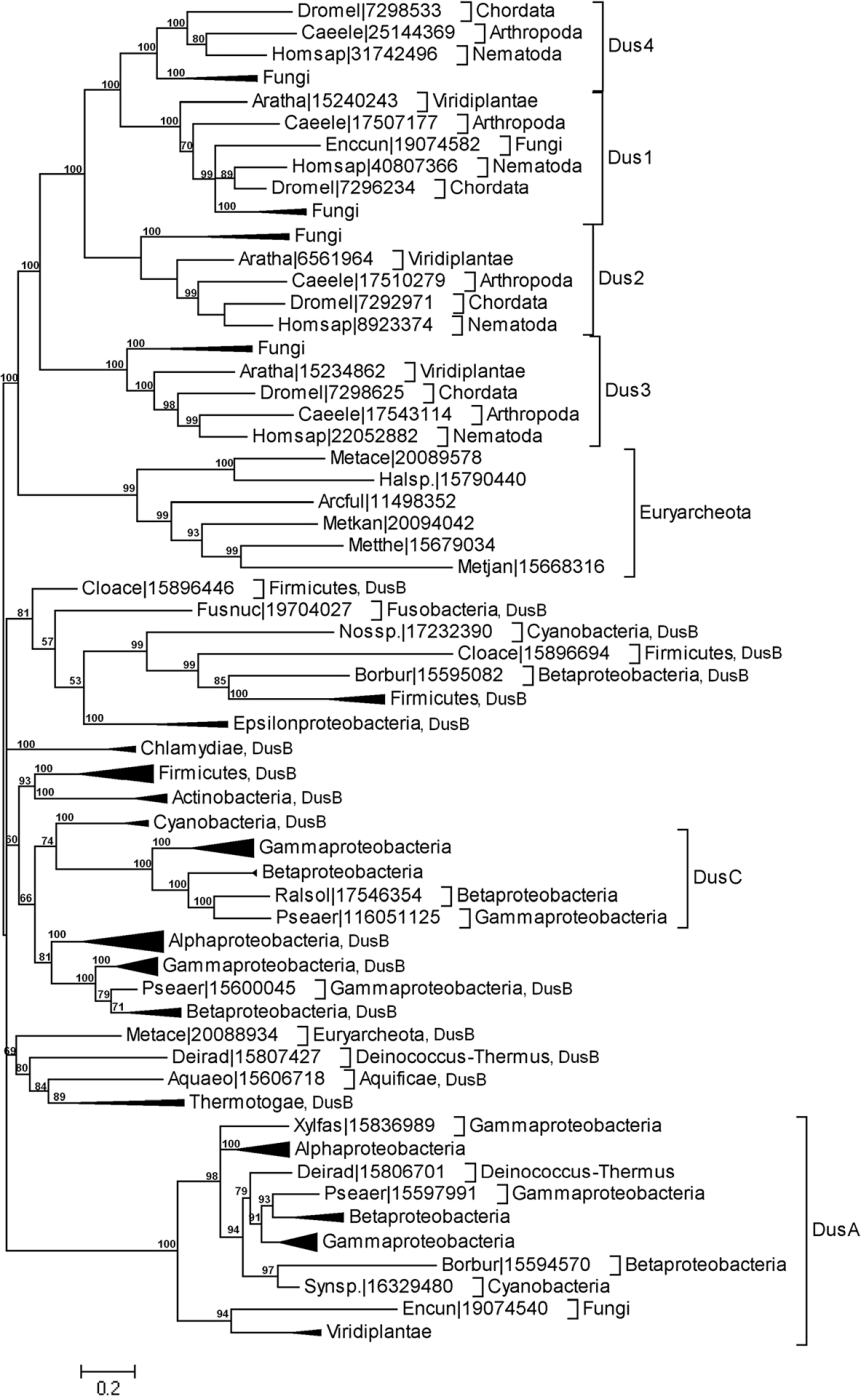
**The Bayesian tree of the DUS family.** Sequences are indicated by the abbreviated genus and species name (e.g. Bacsub for *B. subtilis*) and the NCBI GI number and their phylogenetic origin are indicated. For clarity of the presentation, branches with sequences belonging to the same taxa have been collapsed and are shown as triangles. Values at the nodes indicate the statistical support for the particular branches, according to the bootstrap test.

The Bayesian tree (Figure 5) reveals significant support for main branches, allowing us to resolve the deep branching pattern. The tree supports the division of DUS family into 8 subfamilies revealed by the pairwise clustering of full-length sequences (Dus1, Dus2, Dus3, Dus4, DusA, DusB, DusC and archaeal Dus). DusB seems to be the oldest subfamily consisting of very divergent sequences whose evolution took a very long time. The prokaryotic DUS families DusA, DusB and DusC are more closely related to each other than to the eukaryotic subfamilies Dus1, Dus2, Dus3, and Dus4. The family of archaeal DUS members also forms a separate branch. Such topology of the tree is in agreement with the topology of the “Tree of Life” (three main life taxa Archaea, Bacteria and Eukaryota) and suggests that only one DUS was present in the Last Universal Common Ancestor of all contemporary cellular organisms (LUCA). Thus, all bacterial and eukaryotic DUS subfamilies were created by independent duplications of the ancestral DUS enzyme.

### A speculative scenario of the evolutionary history of the DUS family

The order of branching of eukaryotic DUS subfamilies suggests that Dus3 may be the ancestral eukaryotic enzyme, from which others have been derived by gene duplication, starting with Dus2, and then Dus1 and Dus4. The absence of Dus4 in Viridiplantae can be explained by gene loss. Dus2p and Dus3p are able to modify only a single specific position in tRNA molecules (20 and 47 respectively), in contrast to Dus4p and Dus1p that can act on two neighboring positions in the same tRNA region. This suggests that the specificity towards one target residue may be evolutionally older than the ability to modify several residues in one region, at least in yeast. Thus, the evolution of these enzymes has followed a trend in which new members of the family showed relaxed specificity compared to their ancestors.

The phylogeny of bacterial DUS families is more complex. DusB appears in nearly all Bacteria, which suggest that it might have been a bacterial DUS ancestor. The ancestor of the DusC subfamily probably appeared as a result of DusB duplication shortly after the divergence of main Proteobacteria groups, in a branch leading to β and γ Proteobacteria. The DusA subfamily contains proteins highly similar to each other, but relatively distinct from other subfamilies. We suggest that DusA appeared by duplication of DusB in an ancestor of Proteobacteria. A single representative of DusA in Cyanobacteria was probably transferred there horizontally.

An interesting feature in the DusB subfamily is the appearance of additional paralogous copies of DUS (Figure 4) in *Bacillus subtilis, Clostridium acetobutylicum* and *Nostoc sp*. Functions of these duplicated genes are unknown. It is possible that they acquired specificities for new positions or that they specialized in modifying the same position in a subset of substrates. Unfortunately, little is known about the position of D residues in tRNAs from these organisms. Definitely, the determination of tRNA sequences from additional organisms would dramatically help in the evolutionary studies of RNA modification enzymes, in particular with respect to their sequence specificities.

The DusA subfamily has members not only in Bacteria but also in plants (*Arabidopsis thaliana*; two proteins: At3g63510 and At5g47970) and fungi (*Encephalitozoon cuniculi*). These genes are most likely products of horizontal gene transfers from endocellular bacterial symbionts: from the alpha-proteobacterial ancestor of a mitochondrion to an ancestor of all contemporary Eukaryota, and from the cyanobacterial ancestor of a chloroplast to the ancestor of green plants. The absence of DusA-like members in many eukaryotic genomes can be simply explained by the gene loss following the initial transfer.

For At3g63510 sequence localization predictions according to the methods used (Sherlock [21], WolfPSORT [22], Plant-Ploc [23], Protein-Powler [24], TargetP [25] and Cello [26]) indicated chloroplast localization, which confirmed our hypothesis that this protein has a cyanobacterial origin. Predictions for At5g47970 sequence, however, failed to identify one preferred localization, and predictions for the DusA-like protein from *Encephalitozoon cuniculi* indicated cytoplasmic localization, hence the site of action of these proteins remains to be characterized experimentally.

**Figure 6:**
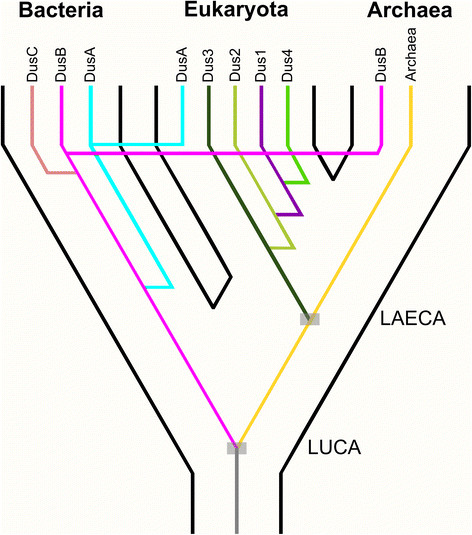
**A speculative scenario of the evolutionary history of the DUS family.** This scenario is based on the assumption that Bacteria, Archaea, and Eukaryota are all monophyletic, that Archaea and Eukaryota are sister lineages, and that the root (corresponding to the LUCA) is located in the branch between Bacteria and the Last Archaeal and Eukaryotic Common Ancestor (LAECA). Three major branches correspond to the three Domains of Life. Each DUS subfamily is represented by a single line of a particular color (e.g. DusB in magenta, DusA in cyan). Bifurcations represent duplications (giving rise to paralogous families), and horizontal lines represent horizontal gene transfers.

Figure 6 shows a proposed scenario of the DUS family evolutionary history, which reconciles the DUS protein tree in the light of the phylogeny of organisms.

### Structural model for the hDus2 protein

In the absence of experimentally determined structure for functionally characterized DUS members (as of 21st June 2011), we constructed a comparative model of a human DUS2 enzyme, to provide a structural platform for the investigation of sequence-structure-function relationships in this family. However, while this article was under review, a crystal structure of dihydrouridine synthase from *Thermus thermophilus* (TthDus) and its complex with tRNA have been determined (PDB code 3b0v) [13]. Hence, we present only a short description of structural model of human Dus in the Results section, and we compare our structural and functional predictions with the independently obtained experimental data in the Discussion section.

**Figure 7:**
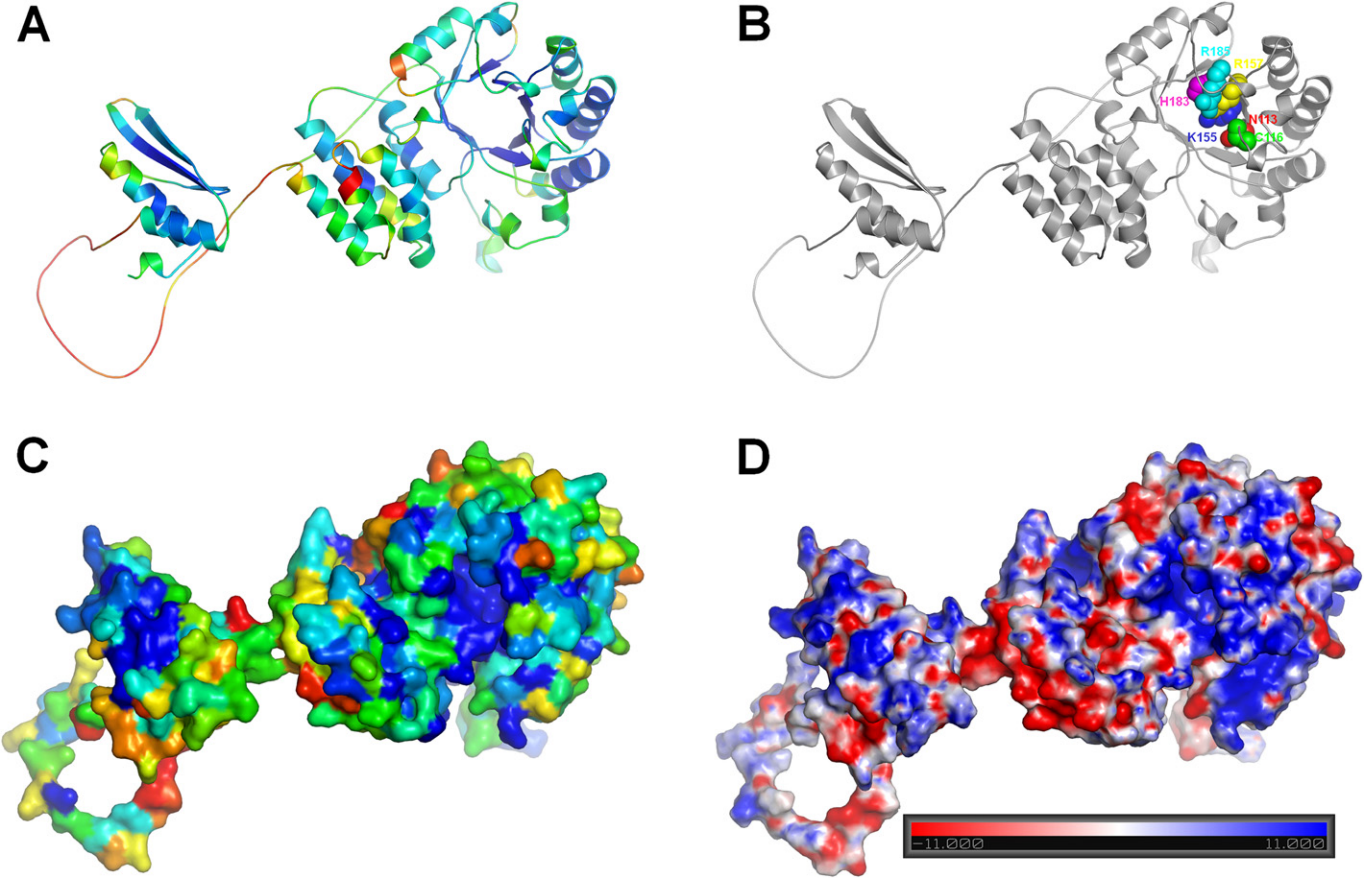
**Structural model of hDus2, a representative of DUS family.** In this model, domains do not interact with each other and are positioned arbitrary (the N-terminal domain on the right, the C-terminal domain on the left), to facilitate visual analysis of binding site. Coordinates are available for download from the FTP server ftp://genesilico.pl/iamb/models/hdus2/ (**A**) Protein shown in a ribbon representation colored according to the estimated accuracy of individual residues calculated by MetaMQAP (Blue indicates low predicted deviation of Cα atoms down to 0 Å, red indicates unreliable regions with deviation > 5 Å; see Methods for details). (**B**) Model backbone shown in the ribbon representation, conserved residues in the active site are labeled and shown in the space-filled representation. For clarity of the presentation, each residue is shown in a different color. (**C**) Protein in a surface representation colored according to sequence conservation in the DUS family, calculated from a multiple sequence alignment using CONSURF [27]. Blue indicates conserved residues, yellow to red indicated variable residues. (**D**) Protein in a surface representation, colored according to the distribution of the electrostatic potential, calculated with the APBS software (Adaptive Poisson-Boltzmann Solver) [28] available via PyMOL [29]. Blue indicates positively charged regions (11 kT), red indicated negatively charged regions (−11 kT). The same view as in the model and its features were visualized with PyMOL [29].

Briefly, the sequence of hDus2 was subjected to structure prediction by the GeneSilico metaserver [18]. Prediction methods identified the catalytic DUS domain (residues 1 ~ 330), a presumably unstructured linker (residues ~331-367), the dsRBD domain (residues 368–433), and a disordered C-terminal extension (residues 434–489). As a template for the catalytic domain, all protein fold-recognition methods returned the TM0096 structure (a functionally uncharacterized protein we found to be a bona fide DUS member earlier in this study) as the potentially best template [16], despite a relatively low sequence similarity (21% identity) (Figure 7). The model of the catalytic domain was created by iterating the homology modeling procedure (initially based on the raw FR alignments of the hDus2 sequence to the top-scoring TM0096 structure), evaluation of the sequence-structure fit by MetaMQAP and manual realignment in poorly scored regions. The final model was obtained following optimization of three uncertain regions (1–11, 62–78 and 253–266) using *de novo* loop modeling with ROSETTA (see Methods for details). The same approach was used to build a model for the dsRBD for which we used the structure of dsRBD from a hypothetical protein BAB26260 protein from *Mus musculus* (PDB code 1whn), which exhibits 84% sequence identity to the relevant region in hDus2. Based on the available data we were not able to predict the mutual orientation of these two domains and we positioned them arbitrarily with respect to each other. The model of hDus2 covered residues 1–441 (of 493 total). It included the interdomain linker (residues 332–269) in an arbitrary conformation, and it lacked the C-terminal disordered region (residues 442–493).

We mapped information about conserved residues from the multiple sequence alignment of hDus2 homologs containing the DSRM domain onto the structure of hDus2 model (Figure 7C) and predicted that the substrate-binding site of hDus2 is most likely formed by residues that are highly conserved among all DUS family members, which correspond to N113, C116, K155, R157, H183, and R185 in hDus2. In the dsRBD the conserved residues included Y388, T390, D396, R397, F399, and S401, and the fragment with residues 419–429. Such conservation suggests that the dsRBD may be functionally important, and the most obvious hypothesis is that its function is related to RNA binding. At present it is not clear how the dsRBD does participate in tRNA binding and recognition by Dus2 members, in particular compared to those members of the Dus2 family that lack the dsRBD.

### Prediction of ligand binding

As mentioned in the introduction, due to the sequence and structure similarity between DHODs, DHPDHs, and TM0096, it has been suggested that these enzymes may use a similar mechanism of catalysis [10]. The DHPDH mechanism of action has been studied in detail [11]. Pyrimidine binding triggers a conformational change of a flexible active-site loop (residues 666–683, contains conserved residues Leu-Asn-Leu-Ser-Cys-Pro) in the barrel domain, resulting in the placement of a catalytically indispensable cysteine residue (C671) close to the bound substrate. The flexible loop closure is an absolute prerequisite for the catalytic activity, as it not only excludes the surrounding solvent from the active site, but most importantly, also places C671 at the location required to enable the proton transfer to the pirimidine C5 atom. From FMN N5, a hydride is transferred to the uracil C6, and the C5 atom receives a proton from the C671 SH group.

**Figure 8:**
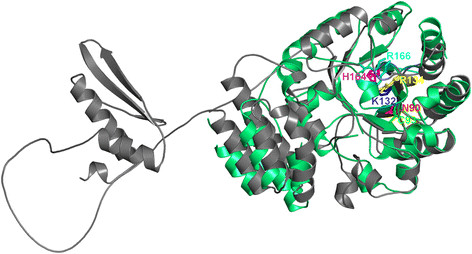
Speculative mechanism of hDus2 action.

Structure comparison of TM0096 and DHODH and DHPDH, indicated that DUS ligands should be positioned very similarly to 5UI and orotate in DHODH and DHPDH complex structures (see Additional 2). Based on comparison of the TM0096 structure with the experimentally determined complexes of these evolutionarily related enzymes, DHODH and DHPDH, we propose the following mechanism of U to D modification in RNA (Figure 8). First, the DUS enzyme containing FMN recognizes and binds the substrate RNA and has the target U positioned in the active site (in the same manner as 5UI and orotate are positioned in DHODH and DHPDH complex structures, respectively). Then, NADPH binds and a proton from FMN N5 is transferred to the C6 atom of the target U. While NADP dissociates, a proton is transferred to the C5 atom of U from the catalytic residue C93. The reduction of the C5-C6 double bond catalyzed by DUS may lead to modification of the product conformation in the binding pocket. Our prediction is in agreement with the one proposed by the Palfey group, who experimentally proved that in Dus2p, reduction of U to D requires two NADPH dependent half-reactions for catalysis (reductive and oxidative) and a cysteine C116 (homologous to C93 in TM0096) is used as a general acid in the reduction of tRNA, as a hydride from reduced FMN is transferred to the uracil ring [30]. Our model of DUS mechanism has been supported by the crystal structure of TthDus in complex with the reaction intermediate, published while this article was under review [13] (see below).

The availability of the TthDus-tRNA complex allowed us to test also the results of the ligand binding prediction. Indeed, as we predicted the position, orientation, and conformation of ligands in DHODH and DHPDH are similar to those of the target nucleotide U20 in the crystal structure of the TthDus-tRNA complex. Thus, our prediction of the protein-ligand complex structure and the mechanism of action of DUS enzymes can be regarded as highly accurate.

### Structural comparison of DUS representatives reveals differences that may be responsible for different substrate specificities

An important question in the comparative analysis of enzymes is the identification of the molecular basis of their different substrate specificities. For DUS, such studies are hampered by the paucity of information about substrate specificities; in particular it is not known whether substrate specificity is conserved within subfamilies. Nonetheless, the comparison of predicted DUS structures, in particular those for members from yeast, together with the analysis of subfamily-specific conserved residues and prediction of RNA-binding residues has revealed potential specificity-determining elements.

In order to identify structural differences between DUS enzymes that may be responsible for their different substrate specificities, we have built homology models for all seven representatives of DUS from yeast (Dus1p, Dus2p, Dus3p, and Dus4p), and *E. coli* (DusA, DusB, and DusC), using the same methodology as for modeling of hDus2. Superposition of these models with annotated conserved and predicted RNA-binding residues (Additional file 3) revealed that while the catalytic cores of these enzymes are very similar, they exhibit variations in internal loops surrounding the active site, and they have very different N and C termini. The terminal extensions are particularly different between eukaryotic enzymes.

According to predictions RNA binding sites might be located in nine regions: I32-R45 (for the purpose of the article called binding region 1, BR1), H62-R73 (BR2), R87-D97 (BR3), D116-L135 (BR4), N147-R162 (BR5), T186-W204 (BR6), P215-Y228 (BR7), D242-P254 (BR8), and A291-F300 (BR9) (numbering of residues as in Dus1p). Dus1p and Dus4p and all bacterial DUS possess predicted RNA-binding residues in all these regions (BR1-BR9) and they cover large part of the protein surfaces. In both Dus1p and Dus4p, predicted RNA-binding surfaces are of a triangular shape with the top vertex formed by a loop (62–73 in Dus1p). In Dus2p and Dus3p, regions corresponding to BR2 and BR3 are not predicted as involved in RNA binding. In Dus3p, BR1 and BR7 are also devoid of predicted RNA-binding residues. In addition, the yeast DUS exhibit different terminal extensions predicted both to be largely disordered (at least in the absence of the RNA substrate) and to contain RNA-binding residues. We predict that it is not the variability of residues in conserved motifs, but rather the length and sequence composition of loops immediately following the conserved motifs (and shaping the surface around the active site) that may contribute most to the substrate specificity of different DUS members. In addition, the highly divergent termini are likely to be involved in sequence-nonspecific interactions with the RNA substrates in such a way that they facilitate the binding of tRNA in some orientations (e.g. by electrostatic interactions) and/or prevent others (e.g. by steric exclusion). These predictions remain to be tested experimentally.

## Conclusions

### Evolution of DUS

We have carried out extensive bioinformatics analyses aimed at comprehensive classification of the DUS family and making functional predictions for the previously uncharacterized proteins. Our results revealed all members of the DUS family, which can be subdivided into 8 subfamilies (DusA, DusB, DusC, Dus1, Dus2, Dus3, Dus4 and archaeal Dus). The results of DUS family clustering combined with the phylogenetic analysis suggest that members of KOG2335 encode proteins from three different subfamilies: DusA, Dus1 and Dus4 (3, 7, and 5 members, respectively). Similarly, COG0042 consists of members from four subfamilies: DusA, archaeal Dus, DusC, and DusB (18, 6, 9, and 41 members, respectively). Furthermore, members of KOG2333 that have been annotated as “uncharacterized conserved proteins of unknown function” belong to the Dus3 subfamily, whereas members of KOG2334 correspond to the Dus2 family. Additionally, based on clustering, a new subfamily of archaeal Dus have been classified which should be from now on included in DUS classification and analysis. The results of our analysis suggest that the groups defined earlier in the COG/KOG database may require revision. We have also analyzed the phylogenetic distributions of DUS family members and inferred their evolutionary tree. The most important conclusion of this part of our work is that LUCA possessed one ancestral DUS enzyme, which underwent independent duplications in Eukaryota and Bacteria, but not in Archaea.

The refined grouping of DUS enzymes into orthologous and paralogous branches provides a framework to study the functional differences among these proteins, in particular their different substrate specificities. Our results will enable easier classification of new DUS members identified in the future.

### Validation of the predictions in the light of the independently determined crystal structure

**Figure 9:**
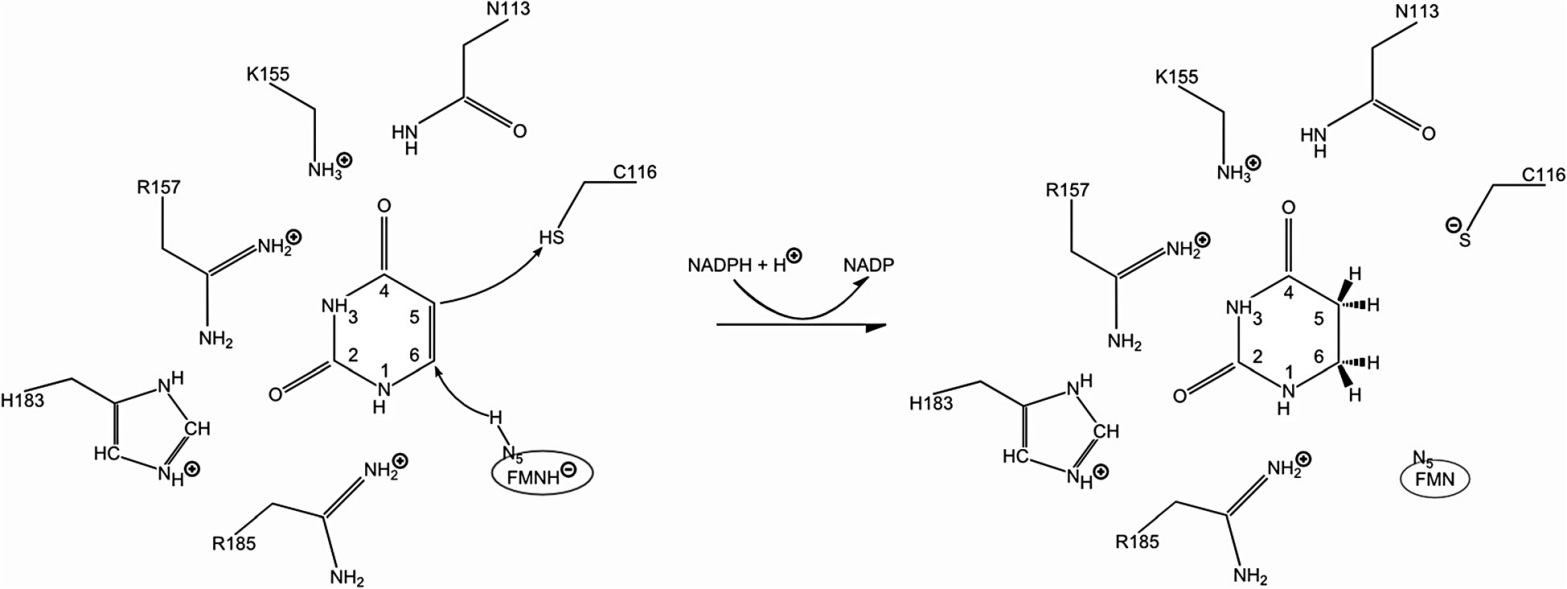
**Structural comparison of hDus2 model and the crystal structure of TthDus.** In the hDus2 model, domains do not interact with each other and are positioned arbitrary (the N-terminal domain on the right, the C-terminal domain on the left), to facilitate visual analysis of the binding site. For both structures the backbones are shown in the ribbon representation (hDus2 model in grey, TthDus in green), conserved residues in the active site are shown in the sticks representation and labeled (numbering is according to crystal structure only). For clarity of the presentation, each residue is shown in a different color (color scheme as in Figure 7).

Comparison of our model with the independently determined crystal structure of TthDus [13] (Figure 9) revealed that we correctly predicted the α/β barrel and helix bundle domains and regions where they bind RNA. The model is particularly accurate in regions responsible for catalysis. The RMSD value between the corresponding parts of the model and the crystal structure (catalytic domain and helix bundle) is very low, only 1.4 Å. We also predicted correctly all key residues involved in catalysis and RNA binding, including N113 (N90 in TthDus), C116 (C93), K155 (K132), R157 (R134), H183 (H164) and R185 (R166) residues. The essential character of these residues has been shown by a series of alanine substitutions done for the TthDus enzyme [13].

### Proposed mechanism of DUS inhibition

The human hDus2 enzyme has been suggested as a possible target for cancer therapy. The knowledge of DUS-ligand interactions and of the catalytic mechanism may help in the development of inhibitors of hDus2. Our homology model of hDus2 enzyme may aid in the efforts towards development of inhibitors for this enzyme. It must be emphasized that DUS-specific inhibitors should be inactive against DHPDH and other related enzymes with a similar structure and mechanism of action in order to reduce their toxicity against human cells. It was shown that 5-iodouracil (5 IU) is an inhibitor of DHPDH activity [31]. NADPH-dependent reduction of 5 IU leads to the formation of 5-iodo-5,6-dihydrouracil, which is released from the active site, but then re-binds, and causes covalent modification of C671, which ultimately blocks the enzyme activity [11]. Derivatives of 5 IU, in particular variants with 5′ and/or 3′ extensions that would prevent binding to DHPDH and other related human enzymes and would e.g. mimic phosphate groups and increase the affinity towards DUS, might be thus potential candidates for inhibitors against hDus2.

## Methods

### Sequence database searches, multiple alignments and comparison of sequence-structure profile HMMs

A set of known members of the DUS family (DusA, NCBI Gene Identification [GI] number: 85676801, DusB GI: 85676051, DusC GI: 85675254 from *Escherichia coli* and Dus1p GI: 6323560, Dus2p GI: 6324342, hDus2 GI: 8923374, Dus3p GI: 85666121, Dus4p GI: 6323437 from *Saccharomyces cerevisiae*) were used as queries in PSI-BLAST [14] searches of the non-redundant (nr) version of the NCBI sequence database (as of 2011) with the expectation (e) value threshold for the retrieval of related sequences set to 10^-25^.

### Sequence clustering

To visualize pairwise similarities between and within protein of DUS homologs we used CLANS (CLuster ANalysis of Sequences), a Java program that applies version of the Fruchterman-Reingold graph layout algorithm [15]. CLANS uses the P-values of high-scoring segment pairs (HSPs) obtained from an N x N BLAST search, to compute attractive and repulsive forces between each sequence pair in a user-defined dataset. 3D or 2D representation is achieved by having points corresponding to sequences randomly positioned in space. The points are then moved within this environment according to the force vectors resulting from all pairwise interactions, the forces are recalculated after the move, and the process is repeated to convergence.

### Sequence editing

All sequences classified as members of the DUS family were aligned using ClustalX [17]. Incomplete sequences were discarded (if the deletion spanned > 30% of the alignment) or repaired using amino acid sequences predicted from the available DNA sequences of the corresponding genes. Manual adjustments were introduced into the alignment to preserve the continuity of secondary structure elements, either observed in crystal structures or predicted computationally (see Protein Structure Prediction).

### Phylogenetic analyses

The phylogenetic tree of the DUS family was inferred for 98 representative members of COG0042 and KOGs 2333, 2334 and 2335 using the alignment of a complete catalytic domain. A Minimum Evolution (ME) analysis carried out with MEGA 4 [32] (with pairwise gaps deletion and either Dayhoff or JTT matrices) were not sufficient to infer a tree with pre-defined subfamilies grouped into monophyletic branches, thus the Bayesian analysis was attempted. Phylogenetic trees were calculated, using the JTT model of substitutions and pairwise deletions, with the initial tree calculated by the NJ method and the Closest Neighbor Search option set to level = 2. The stability of individual nodes was calculated using the bootstrap test (1000 replicates) and additionally confirmed by the interior branch test – in all trees, for all branches with bootstrap support >50%, the ITB support was equal or higher.

MrBayes MPI version 3.1.2 [20] was used to carry out a Bayesian analysis of sequences data. All analyses were performed using the sequence alignment for family representatives, with a gamma distribution of substitution rates, using the default approximation of four rate classes for each. Preliminary runs with MrBayes using a mixture of model priors demonstrated conclusively that substitution rates from the WAG/BLOSUM matrices [33] provided best fit to the sequence data. Therefore, the WAG/BLOSUM model was used to provide substitution priors for the sequence partition of the data. A Metropolis-coupled Markov-chain Monte-Carlo analysis was performed with 2 million generations, two runs and eight chains (four per run). The Markov chain was sampled every 100 generations. Convergence of runs was confirmed by average standard split deviation factor that falls under the recommended value of 0.01. The final tree was obtained after removing the first 25% of samples.

### Protein structure prediction

Protein sequences were submitted to the GeneSilico metaserver, which is a gateway to a large number of third-party methods that facilitates comparison and interpretation of predictions made by different algorithms [18]. In particular, the metaserver was used for secondary structure prediction and for protein fold-recognition (i.e. alignment of protein sequence to proteins with experimentally determined structures that can be used as templates for modeling). Fold-recognition alignments reported by primary methods were compared, evaluated, and ranked by the PCONS method [34]. PCONS score >1 in general indicates estimation that the protein fold has been correctly guessed by FR methods. However, lower scores do not necessarily exclude correct predictions, in particular for folds with strongly diverged members. In such cases, a good estimator of prediction quality is the number of occurrences of a given fold at the top positions of the PCONS ranking. PCONS has traditionally performed very well in all editions of CASP and other benchmarks [35].

The fold-recognition alignments of hDus2 and the top-scoring templates (PDB codes 1vhn and 1whn for N-terminal and C-terminal hDus2 domains, respectively) were used as a starting point for modeling of hDus2 tertiary structure comprising cycles of model building by Modeller [36], evaluation by MetaMQAP [37], realignment in poorly scored regions as long as manual alignment changes does not improve model quality. Uncertain regions (residues 1–11, 62–78, 253–266) were modeled *de novo* using ROSETTA [38] in the context of ‘frozen’ remainder of the hDus2 model. Briefly, fragment selection based on profile-profile and secondary structure comparison with the ROSETTA database was performed and 3 and 9 amino acids fragments lists were generated for the remodeled regions. The fragment assembly was performed with default options and medium level of side chains rotamers optimization. The set of 5000 preliminary models (decoys) of was clustered and representatives of 3 largest clusters were selected as the final structures for evaluation.

### Protein model evaluation

For evaluation of models we used two Model Quality Assessment Programs (MQAPs): MetaMQAP [37] and PROQ [39]. It must be emphasized that MQAP scores only predict the deviation of a model from the real structure (the real deviation can be calculated only by comparison to the real structure, which of course is not available). Thus, the scores reported in this work that indicate e.g. ‘very good models’, must be interpreted as estimations or predictions that our models are ‘very good’, and not as ultimate validation of the model quality. However, it should be mentioned that both PROQ and MetaMQAP performed very well in CASP and in independent benchmarks and can be regarded as robust predictors.

### Prediction of intrinsically disordered residues

Predictions of intrinsically disordered residues were made using MetaDisorder (http://iimcb.genesilico.pl/metadisorder/; [40], a meta-method which combines the predictions of the following primary methods: DisEMBL [41], DISPROT(VSL2) [42], GlobPlot [43], IUPred [44], PDISORDER (SoftBerry, http://linux1.softberry.com/berry.phtml), POODLE-S [45], POODLE-L [46], PrDOS [47], Prosat (http://glaros.dtc.umn.edu/gkhome/node/456) and RONN [48].

### Prediction of RNA-binding residues

Prediction of RNA-binding residues for protein sequences was made using a specialized meta-predictor [49] based on three sequence-based primary predictors that ranked highest in our tests (PiRaNhA [50], PPRInt [51], and BindN + [52]).

## Abbreviations

DUS, dihydrouridine synthase; aa, amino acid(s); e-value, expectation value.

## Competing interests

Authors declare that they have no competing interests.

## Authors' contributions

JMK carried out sequence database searches, structure predictions, phylogenetic analyses, drafted the manuscript and prepared the figures. AC participated in homology modeling. JMB designed and coordinated the study, and edited the manuscript. All authors analyzed and interpreted the data. All authors have read and approved the final manuscript.

## Supplementary Material

Additional file 1 **MSA_tree.aln.** Multiple sequence alignment of all DUS sequences from the COG/KOG database used to calculate a phylogenetic tree. Sequences are denoted by the COG/KOG number, species’ name (six-letter abbreviation for genus or species e.g. Bacsub for *B.subtilis*), followed by the protein name (if assigned) or the sequence name from COG/KOG database. The variable termini and non-conserved insertions have been removed. Click for file here

Additional file 2 **structures_superposition.pse.** Superposition of DHPDH, DHODH, TthDus and hDus2 model structures. Active site residues, cofactors and ligands are shown in sticks representation and each structure is shown in a different color (DHPDH in blue, DHODH in magenta, TthDus in green and hDus2 in grey). The file is a PyMOL session. Click for file here

Additional file 3 **Dus_models_RNAbindingSites.pse.** Superposition of structural models of DUS representatives. All proteins are shown in surface representation and putative RNA binding sites are highlighted by different colors. Additionally each protein is shown in surface representation and colored by residue conservation within closest homologs calculated by the Consurf method (the color pattern is the same as in Figure 7). The file is a PyMOL session. Click for file here
